# Ni Center Coordination Reconstructed Nanocorals for Efficient Water Splitting

**DOI:** 10.1002/advs.202205605

**Published:** 2022-11-16

**Authors:** Tianyi Xu, Dongxu Jiao, Manman Liu, Lei Zhang, Xiaofeng Fan, Lirong Zheng, Weitao Zheng, Xiaoqiang Cui

**Affiliations:** ^1^ State Key Laboratory of Automotive Simulation and Control School of Materials Science and Engineering Key Laboratory of Automobile Materials of MOE Jilin Provincial International Cooperation Key Laboratory of High‐Efficiency Clean Energy Materials Electron Microscopy Center Jilin University Changchun 130012 P. R. China; ^2^ College of Chemistry Jilin University 2699 Qianjin Street Changchun 130012 P. R. China; ^3^ Beijing Synchrotron Radiation Facility Institute of High Energy Physics Chinese Academy of Sciences Beijing 100049 P. R. China

**Keywords:** coordination reconstruction, electrocatalyst, nanocoral structures, Ni center, overall water splitting

## Abstract

Efficient electrocatalytic reactions require a coordinated active center that may provide a properly reaction intermediates adsorption in water splitting. Herein, a Ni active center coordination reconstruction method achieved by multidimensional modulation of phase transition, iodine coordination, and vacancy defects is designed and implemented. This coordination reconstruction results in the successful synthesis of Ni_5_P_4−_ *_x_* I*_x_*/Ni_2_P nanocorals that show outstanding bifunctional catalytic activity due to deep optimization of the adsorption energy. The overpotentials of hydrogen evolution reaction and oxygen evolution reaction at 10 mA cm^−2^ are 46 and 163 mV, respectively. Only 1.46 V is required to drive alkaline overall water splitting. Novel coordination environment is investigated by electron paramagnetic resonance spectroscopy and extended X‐ray absorption fine structure spectroscopy. A 4D integrated material design strategy of “thermodynamic stability‐electronic properties‐charge transfer‐adsorption energy” for water‐splitting catalysts is proposed. This coordination reconstruction concept and material design method provide new perspectives for the research of novel catalysts.

## Graphical abstract

Ni active center coordination reconstruction method is achieved by multidimensional modulation of phase transition, iodine coordination, and vacancy defects. This coordination reconstruction results in the successful synthesis of Ni_5_P_4−_ *_x_* I*_x_*/Ni_2_P nanocorals that show outstanding bifunctional catalytic activity due to deep optimization of the adsorption energy, derives a 4D integrated material design strategy of “thermodynamic stability‐electronic properties‐charge transfer‐adsorption energy”.

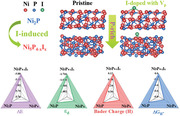

## 1 Introduction

Water splitting is an important way to produce high purity clean hydrogen that may resolve the current environmental pollution and energy demand.^[^ ^1^ ^]^ Water splitting is divided into two half‐reactions of hydrogen evolution reaction (HER) and oxygen evolution reaction (OER),^[^ ^2^ ^]^ in which precious metal catalysts are used to reduce the reaction potential. However, the high price and scarce reserves of precious metals hinder the industrial application of water splitting.^[^ ^3^ ^]^ Recently, developing of transition metal‐based replacement of noble metal‐based catalysts has been the focus of the new energy research, such as transition metal carbides,^[^ ^4^ ^]^ nitrides,^[^ ^5^ ^]^ sulfides,^[^ ^6^ ^]^ phosphides,^[^ ^7^ ^]^ alloys,^[^ ^8^ ^]^ etc.

Transition metal phosphides showed promising potential because of their high electron conductivity, corrosion resistance, and inherent activity.^[^ ^9^ ^]^ The abundant valence states of transition metals and phosphorus derive two types of transition metal phosphides (M*_x_* P*_y_*) of metal‐rich phosphides and P‐rich phosphides.^[^ ^10^ ^]^ Different stoichiometric ratios bring about great differences in crystal structure and bonding.^[^ ^10^, ^11^ ^]^ The M—M bond in the metal‐rich phosphide enables the material metallic and corrosion resistant.^[^ ^12^ ^]^ Prominent nucleophilicity of the metal center facilitates water adsorption but lacks proton trapping ability.^[^ ^13^ ^]^ P‐rich phosphides provide abundant proton capture sites, while too many P atoms also bring excessive hydrogen adsorption.^[^ ^14^ ^]^ Element doping is widely used to enhance the performance of both types of transition metal phosphides.^[^ ^15^ ^]^ Deng et al. reported an Fe‐doped Ni_2_P nanoparticle, and the doping of Fe element modulates the electronic structure and microscopic morphology of the catalyst.^[^ ^16^ ^]^ Wang et al. proposed an N‐doped CoP_2_ to improve HER performance by N‐induced lattice contraction.^[^ ^17^ ^]^ The doping of heteroatoms enables the optimization of the catalytic performance. However, these doping strategies may only change the localized coordination environment of the active center due to the preservation of the original crystal structure. Deep optimization of hydrogen absorption/desorption requires a scientifically challenging design strategy for coordination reconstruction of the active center.

Herein, we propose a novel strategy for the coordination reconstruction of active centers. Our results show that the doping of iodine atoms changes the original thermodynamic stability and induces the transformation of Ni_2_P into Ni_5_P_4−_ *_x_* I*_x_* phase with P vacancies. Deep optimization of intermediate absorption/desorption was obtained by multidimensional coordination reconstruction of new phases, iodine coordination, and vacancy defects. We reveal a 4D integrated material design strategy of “thermodynamic stability‐electronic properties‐charge transfer‐adsorption energy” (TECA) by considering the correlation between the feasibility of synthesis and theoretical performance. Ni_5_P_4−_ *_x_* I*_x_* nanocoral was successfully synthesized by phosphorization under iodine vapor atmosphere, which shows unprecedented performances for water splitting. In alkaline media, the overpotentials of HER and OER at 10 mA cm^−2^ are 46 and 163 mV, respectively. Only an ultralow cell voltage of 1.46 V is required for water splitting at 10 mA cm^−2^. These excellent performances are ascribed to the novel coordination reconstruction that was fully investigated by electron paramagnetic resonance (EPR) spectroscopy and extended X‐ray absorption fine structure (EXAFS) spectroscopy. The active center coordination reconstruction concept and TECA design method explore new avenues for energy material innovation.

## 2 Results and Discussion

**Figure 1:**
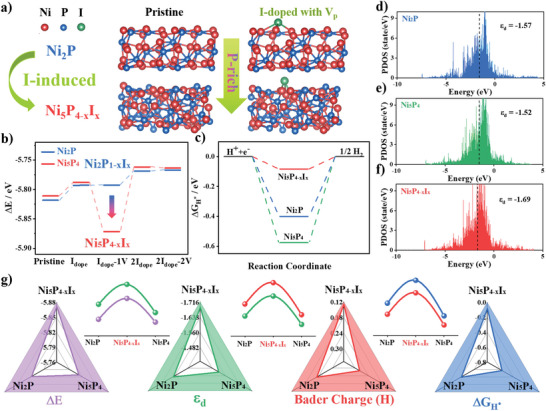
a) Optimized theoretical structural models for Ni_2_P, Ni_2_P_1−_ *_x_* I*_x_*, Ni_5_P_4_, and Ni_5_P_4−_ *_x_* I*_x_*. b) Cohesive energies (Δ*E*) of Ni_2_P and Ni_5_P_4_ based on structures of pristine, I‐doped, I‐doped with 1 P vacancy (1 V), 2I‐doped, 2I‐doped with 2 P vacancies (2 V). c) Free energy diagrams for H adsorption on catalytic surfaces (Δ*G*_H*_). d–f) Ni 3d PDOS of Ni_2_P, Ni_5_P_4_, and Ni_5_P_4−_ *_x_* I*_x_*_._ g) Radar plots of Ni_2_P, Ni_5_P_4_, and Ni_5_P_4−_ *_x_* I*_x_*_,_ correspond to four indicators: Δ*E* (purple), *ε*_d_ (green), Bader charge of H* (red) and Δ*G*_H*_ (blue), and the correlation trend of the four indicators.

Density functional theory was used for simulation of the coordination reconfiguration and the performance evaluation. **Figure** **1**a shows the atomic models of I‐doping‐induced crystal structure transition from Ni_2_P to Ni_5_P_4−_ *_x_* I*_x_*. Calculations of the cohesion energy (Δ*E*) were used to evaluate the feasibility of this thermodynamic transition (Figure 1b). Ten structural models were constructed by considering the I‐doping and consequently induced vacancies from the pristine Ni_2_P and Ni_5_P_4_ (Figure 1b and Figures S1 and S2, Supporting Information). Pristine Ni_2_P and I‐doped Ni_2_P show favorable formation energies than the pristine Ni_5_P_4_ and I‐doped Ni_5_P_4_, which is in good agreement with the fact that Ni_2_P is normally synthesized in experiments.^[^ ^18^ ^]^ However, single site iodine doping with one vacancy reverses the original thermodynamic trend and makes Ni_2_P_1−_ *_x_* I*_x_* spontaneously transform into Ni_5_P_4−_ *_x_* I*_x_*. With increasing I‐doping content and vacancies, the formation advantage of Ni_5_P_4−_ *_x_* I*_x_* disappears. Therefore, the doping of iodine atoms in Ni_2_P will induce the formation of Ni_5_P_4−_ *_x_* I*_x_* with vacancies. The H adsorption free energy (Δ*G*_H*_) of this new structure is studied to evaluated its theoretical catalytic activity.^[^ ^19^ ^]^ As shown in Figure 1c, Ni_5_P_4−_ *_x_* I*_x_* shows a Δ*G*_H*_ of 0.08 eV that is closer to 0 compared to Ni_2_P and Ni_5_P_4_, suggesting that the coordination reconstruction of the Ni site benefits the HER performance. We used electronic analysis to further investigate the regulating mechanism between the reconstituted Ni sites and hydrogen adsorption.^[^ ^20^ ^]^ Charge density difference shows the redistribution of electrons due to iodine doping, phase transition, and vacancy defects (Figure S3, Supporting Information). The charge density around the Ni active site shows significant variation, which is favorable to improve the catalytic activity. Ni 3d partial density of states (PDOS) reveals that the coordination reconstruction moves the d‐band center away from the Fermi energy level, which compensates for the original over‐adsorption^[^ ^21^ ^]^ (Figure 1d–f). The crystal orbital Hamilton population analysis also demonstrates that the interaction between H and Ni centers is weakened in Ni_5_P_4−_ *_x_* I*_x_* (Figure S4, Supporting Information). Previous researches on material simulations and designs have focused on adsorption energy Δ*G* and d‐band centers.^[^ ^22^ ^]^ However, few of them set up a synergistic correlation between the thermodynamic feasibility of synthesis and their theoretical catalytic properties. We present Δ*E*, *ε*_d_, Bader charge of H*_,_ and Δ*G*_H*_ as four indicators through radar plots as shown in Figure 1g. Ni_5_P_4−_ *_x_* I*_x_* exhibits clear advantages in all these indicators. Interestingly, the fluctuating trends of the four indicators show a remarkable consistency in describing the energy and electronic properties of Ni_2_P, Ni_5_P_4_, and Ni_5_P_4−_ *_x_* I*_x_*. On the basis of these results, we reveal a 4D integrated material design strategy of TECA. To begin with, this design strategy focuses on the thermodynamic differences in formation energies and selects structures with formation advantages. Then the electronic properties of the new structures and the charge transport with the catalytic intermediates are explored. Further analysis of the adsorption energies completes a material simulation design that can be accurately implemented in experiments.

**Figure 2:**
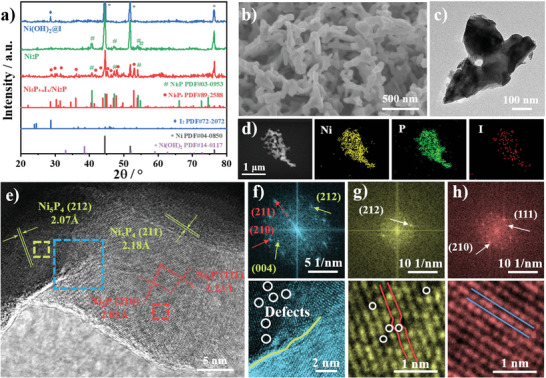
a) X‐ray diffraction patterns of Ni_2_P/NF, Ni_5_P_4−_ *_x_* I*_x_*/Ni_2_P/NF, and Ni(OH)_2_@I/NF. b,c) SEM, TEM images of Ni_5_P_4−_ *_x_* I*_x_*/Ni_2_P. d) EDX mapping images of Ni, P, and I elements for Ni_5_P_4−_ *_x_* I*_x_*/Ni_2_P. e) HRTEM image of Ni_5_P_4−_ *_x_* I*_x_*/Ni_2_P. f–h) FFT and IFFT of the interface (blue), Ni_5_P_4_ (green), and Ni_2_P(red) square in (e).

Ni_5_P_4−_ *_x_* I*_x_* was then experimentally synthesized by introducing iodine vapor into the phosphorization heat treatment. Crystal structures characterization of samples by X‐ray diffractometry is shown in **Figure** **2**a. After the phosphating treatment accompanied by iodine vapor, the synthesized nickel phosphide consists of two phases of Ni_5_P_4_ (PDF# 89–2588) and Ni_2_P (PDF# 03–0953), consistent with the phase transition predicted by the theoretical design. In contrast, simple phosphorization in the absence of iodine forms pure Ni_2_P, and merely iodine treatment of Ni(OH)_2_ covers a layer of I_2_ (PDF# 72–2072) on the surface of the precursor. The main component of the mixed phase can be further regulated by temperatures of 250, 350, 450, and 550 °C (Figure S5, Supporting Information). Scanning electron microscopy (SEM) and transmission electron microscopy (TEM) were used to observe morphology and lattice. Figure 2b,c shows that Ni_5_P_4−_ *_x_* I*_x_*/Ni_2_P has a multibranched nanocoral structure with open pores, which facilitates the exposure of more active sites and the desorption of bubbles.^[^ ^23^ ^]^ This structure is not observed in the precursor and comparison samples (Figures S6 and S7, Supporting Information). Morphology of nanocorals varied with heat treatment temperature (Figure S8, Supporting Information). Energy dispersive X‐ray spectroscopy (EDX) shows uniform distribution of Ni, P, and I elements (Figure 2d). High‐resolution TEM (HRTEM) in Figure 2e shows two types of nickel phosphide lattice stripes, where 2.07 and 2.18 Å correspond to the (212) and (211) crystal planes of Ni_5_P_4_, respectively. There are also another set of lattice stripes of 2.01 and 2.23 Å, corresponding to the (210) and (111) crystal planes of Ni_2_P, respectively. Fast Fourier transform (FFT) and inverse fast Fourier transform (IFFT) were performed on the regions inside three boxes in Figure 2e to extract more information about the lattice differences. Two different sets of diffraction spots can be observed more clearly at the junction of the two phases of Ni_5_P_4_ and Ni_2_P (Figure 2f and Figure S9, Supporting Information). As compared with Ni_2_P, Ni_5_P_4_ shows more defect sites, which is caused by the introduction of I, in accordance with the predictions of the theoretical simulations (Figure 2g,h).

**Figure 3:**
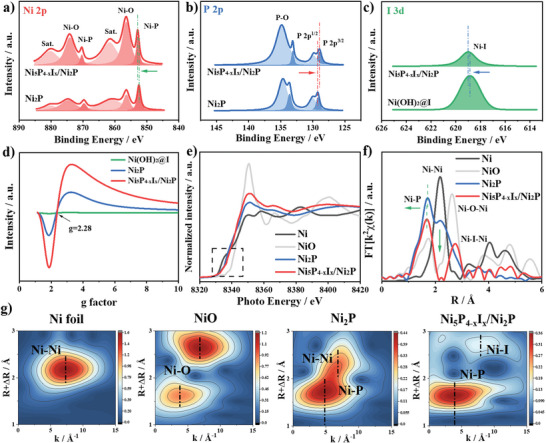
High‐resolution XPS spectra of a) Ni 2p, b) P 2p of Ni_2_P and Ni_5_P_4−_ *_x_* I*_x_*/Ni_2_P. c) I 3d of Ni_5_P_4−_ *_x_* I*_x_*/Ni_2_P and Ni(OH)_2_@I. d) EPR spectra of Ni(OH)_2_@I, Ni_2_P, and Ni_5_P_4−_ *_x_* I*_x_*/Ni_2_P. e) The normalized XANES spectra of the Ni K‐edge and f) Fourier transform of the Ni K‐edge EXAFS of Ni foil, NiO, Ni_2_P, and Ni_5_P_4−_ *_x_* I*_x_*/Ni_2_P. g) Wavelet transformed plots for the *k* ^3^‐weighted EXAFS signals of the Ni K‐edge of Ni foil, NiO, Ni_2_P, and Ni_5_P_4−_ *_x_* I*_x_*/Ni_2_P.

X‐ray photoelectron spectroscopy (XPS) was used to study the state changes of surface chemical elements after coordination reconstruction. As shown in **Figure** **3**a, the peaks at 852.7, 856.3, and 861.5 eV correspond to the Ni—P, Ni—O, and the typical satellite peak of Ni_5_P_4−_ *_x_* I*_x_*/Ni_2_P, respectively.^[^ ^12^, ^14^ ^]^ Compared to the Ni—P bond of Ni_2_P, a high energy shift of 0.3 eV is observed in Ni_5_P_4−_ *_x_* I*_x_*, attributed to more electron transfer in the new phase. Ni(OH)_2_@I has only Ni—O and satellite peaks (Figure S10, Supporting Information). P 2p^3/2^ peak position shifts toward lower binding energy, indicating an increase in electron density around the P site (Figure 3b). Compared to Ni(OH)_2_@I, the Ni—I bond in Ni_5_P_4−_ *_x_* I*_x_*/Ni_2_P has a higher binding energy, implying that the phosphorization promotes the coupling between Ni and I (Figure 3c). The introduction of I is accompanied by a significant increase in the percentage of P elements (Table S1, Supporting Information), which demonstrates the generation of Ni_5_P_4_ phase and changes in the coordination environment of nickel metal. EPR was used to study the electron spin state of the Ni atomic orbitals. A higher signal intensity was observed for Ni_5_P_4−_ *_x_* I*_x_*/Ni_2_P compared to the original Ni_2_P sample (Figure 3d). This stems from the fact that vacancy defects lead to more unpaired electrons.^[^ ^24^ ^]^ This modulation of electron spins is also dependent on the synthesis temperature, among which 350 °C also shows the best (Figure S11, Supporting Information), this is maybe due to the morphological effect.

The coordination of Ni site was further investigated by X‐ray absorption near‐edge structure (XANES) and EXAFS spectroscopy. As shown in Figure 3e, the valence state of Ni in both nickel phosphides is between Ni and NiO. After the iodine‐induced formation of Ni_5_P_4−_ *_x_* I*_x_*, the K‐edge spectrum of Ni shifts to higher energies, which is consistent with the XPS results. The coordination bonding of Ni can be observed more clearly by the R‐space obtained by Fourier transform and extended XANES oscillation functions *k* ^2^ *χ*(*k*) (Figure 3f and Figures S12 and S13, Supporting Information). The shortening of Ni—P bond and the disappearance of Ni—Ni in Ni_5_P_4−_ *_x_* I*_x_* are characteristic of the production of P‐rich phases compared to Ni_2_P.^[^ ^25^ ^]^ The decrease in Ni—P intensity is caused by iodine coordination competition and the presence of defects. The Ni—I can be further corroborated by the comparison sample Ni(OH)_2_@I (Figures S14 and S15, Supporting Information). The wavelet transform (WT) EXAFS contour map shows the appearance of Ni—I bond and the disappearance of Ni—Ni, confirming the coordination reconstruction of the Ni active center (Figure 3g and Figure S16, Supporting Information). Interestingly, the wave vector of Ni—P bond decreases from 4.82 to 3.94 Å^−1^, which implies that the increase of P coordination makes the average atomic number lower.^[^ ^22^, ^25^ ^]^ The above electronic spectroscopic characterization provides sufficient evidence for the coordination environment reconstruction of the nickel center, which is consistent with the theoretical prediction.

**Figure 4:**
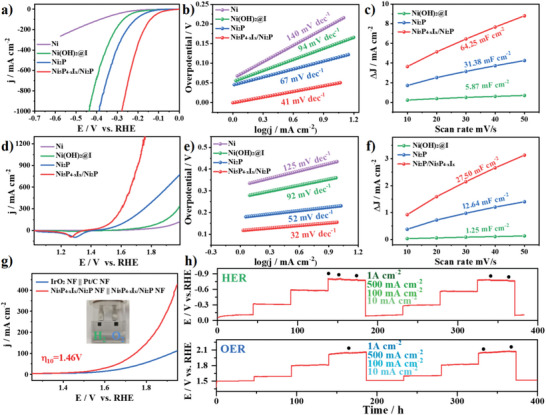
Electrochemical performances of a) HER polarization curves of the Ni_5_P_4−_ *_x_* I*_x_*/Ni_2_P recorded at a scan rate of 2 mV s^−1^, along with Ni_2_P, Ni(OH)_2_@I, and NF for comparison. b) Tafel slopes derived from HER polarization curves. c) Double‐layer capacitances (*C*_dl_) for HER comparison of Ni(OH)_2_@I and Ni_2_P. d) OER polarization curves and e) corresponding Tafel slopes for the above four electrocatalysts. f) Double‐layer capacitances (*C*_dl_) for OER comparison of Ni(OH)_2_@I and Ni_2_P. g) Two electrode polarization curves with a scan rate of 2 mV s^−1^ for bifunctional Ni_5_P_4−_ *_x_* I*_x_*/Ni_2_P /NF, compared with the IrO_2_ NF || Pt/C NF couple (inset is a digital image of water splitting). h) Long‐term HER and OER stability tests for Ni_5_P_4−_ *_x_* I*_x_*/Ni_2_P at step current densities of 10100, 500, and 1000 mA cm^−2^.

Electrocatalytic performances of Ni_5_P_4−_ *_x_* I*_x_*/Ni_2_P, Ni_2_P, Ni(OH)_2_@I, and Ni were tested in 1 m KOH electrolyte. As shown in **Figure** **4**a, Ni_5_P_4−_ *_x_* I*_x_*/Ni_2_P exhibits excellent HER activity, requiring only 45 mV to reach a current density of 10 mA cm^−2^ compared to Ni_2_P (113 mV), Ni(OH)_2_@I (148 mV), and Ni (200 mV). The corresponding Tafel slope is only 41 mV dec^−1^, indicating that the coordination reconstruction accelerates the reaction kinetics (Figure 4b). Ni_5_P_4−_ *_x_* I*_x_*/Ni_2_P has a smaller Nyquist fit curve in electrochemical impedance spectroscopy tests (Figure S17, Supporting Information), representing the advantage of charge transport in catalytic processes. The electrochemical active surface area was evaluated by double‐layer capacitance. The significant increase in active area is closely related to the specific nanocoral morphology (Figure 4c and Figure S18, Supporting Information). Compared with the HER performance of the materials synthesized at different temperatures, Ni_5_P_4−_ *_x_* I*_x_*/Ni_2_P‐350 showed the best HER activity (Figure S19, Supporting Information). Probing the surface adsorption properties by Brunauer–Emmett–Teller, Ni_5_P_4−_ *_x_* I*_x_*/Ni_2_P has a significantly larger specific surface area and optimized pore size and volume compared to Ni_2_P (Figures S20 and S21, Supporting Information). The nanocoral morphology exhibits superhydrophilic characteristics in the wettability test (Figure S22, Supporting Information), which is very favorable for the rapid desorption of bubbles in the catalytic process.

The Ni_5_P_4−_ *_x_* I*_x_*/Ni_2_P also has excellent OER performance, with overpotentials as low as 163 mV at a current density of 10 mA cm^−2^ (Figure 4d). The corresponding Tafel slope is only 32 mV dec^−1^ (Figure 4e). Ni_5_P_4−_ *_x_* I*_x_*/Ni_2_P also shows larger electrochemically active area and lower charge transfer resistance (Figure 4f and Figures S23 and S24, Supporting Information). Considering the excellent HER and OER performance, Ni_5_P_4−_ *_x_* I*_x_*/Ni_2_P is used as both cathode and anode for overall water splitting (OWS). The cell voltage required for 10 mA cm^−2^ in 1 m KOH electrolyte is 1.46 V, which is a significant advantage over the current commercial catalyst (−) Pt/C/NF || IrO_2_/NF (+) (1.55 V) (Figure 4h). The HER, OER, and OWS catalytic performance exhibited by Ni_5_P_4−_ *_x_* I*_x_*/Ni_2_P is superior to most of the catalysts reported to date (Table S2, Supporting Information). These excellent OER and OWS performances of Ni_5_P_4−_ *_x_* I*_x_*/Ni_2_P are also confirmed by the theoretical calculations. The OER free energies of Ni_2_P, Ni_5_P_4_, and Ni_5_P_4−_ *_x_* I*_x_* are calculated (Figure S25, Supporting Information). Ni_5_P_4−_ *_x_* I*_x_* has more advantages in the rate‐determining step of O*‐OOH*, which is consistent with the experimental results. The dissociation energy of water during alkaline HER of three materials is also compared (Figure S26, Supporting Information). Ni_5_P_4−_ *_x_* I*_x_* requires a lower energy potential barrier and has an outstanding advantage of water splitting. Stability is another important parameter for assessing catalyst performance.^[^ ^26^ ^]^ Ni_5_P_4−_ *_x_* I*_x_*/Ni_2_P maintained high HER activity for 382 h in step current tests up to 1 A cm^−2^ (Figure 4h and Figure S27, Supporting Information). Ni_5_P_4−_ *_x_* I*_x_*/Ni_2_P maintains OER stability for up to 400 h at step currents up to 1 A cm^−2^ (Figure 4h and Figure S28, Supporting Information). This shows potential for industrial applications. Therefore, the high activity, high stability, and low cost of the coordination reconstructed nanocorals are of great importance for the technological innovation of the water electrolysis industry.

## 3 Conclusion

In summary, we report an active center coordination reconstruction strategy by regulating the thermodynamic formation energy through introducing iodine atoms, resulting in phase transition and vacancy defects. The active center coordination environment is precisely controlled by temperature and large negative doping atoms and fully verified by EPR and EXAFS techniques. Ni_5_P_4−_ *_x_* I*_x_*/Ni_2_P exhibits unprecedented superior water‐splitting performances to most of the reported materials. The overpotential at 10 mA cm^−2^ is 46 mV for HER and 163 mV for OER. Only 1.46 V is required to drive 10 mA cm^−2^ overall water splitting in an alkaline electrolyzer. This coordination reconstruction concept and material design method widens the path for energy material optimization and upgrading.

## Conflict of Interest

The authors declare no conflict of interest.

## Supporting information

Supporting Information

## Data Availability

The data that support the findings of this study are available from the corresponding author upon reasonable request.
