# Supplementary material for: Ni Center Coordination Reconstructed Nanocorals for Efficient Water Splitting
Source: Adv Sci (Weinh). 2022 Nov 16;10(4):2205605. doi: 10.1002/advs.202205605 (PMC9896050; doi:10.1002/advs.202205605)
Supplement: Supplementary file 1 — Supporting Information [file ADVS-10-2205605-s001.pdf]

Supporting Information

Ni center coordination reconstructed nanocorals for efficient water splitting

Tianyi Xu [1], Dongxu Jiao [1], Manman Liu [1], Lei Zhang [2], Xiaofeng Fan [1], Lirong Zheng [3], Weitao Zheng*[1] and Xiaoqiang Cui*[1]

[1] State Key Laboratory of Automotive Simulation and Control, School of Materials Science and Engineering, Key Laboratory of Automobile Materials of MOE, Jilin Provincial International Cooperation Key Laboratory of High-Efficiency Clean Energy Materials, Electron Microscopy Center, Jilin University, Changchun 130012, China

[2] College of Chemistry, Jilin University, 2699 Qianjin Street, Changchun 130012, China

[3] Beijing Synchrotron Radiation Facility, Institute of High Energy Physics, Chinese Academy of Sciences, Beijing 100049, China

*Corresponding Author: Xiaoqiang Cui; Weitao Zheng;

E-mail: xqcui@jlu.edu.cn (X. Q. Cui); wtzheng@jlu.edu.cn;

Table of Contents

Experimental Methods

1. ***Materials and chemicals.. P3.***
2. ***Synthesis methods...P3-4.***
3. ***Materials characterization...P4.***
4. ***Electrochemical Measurements...P5***
5. ***DFT calculations..P5-6.***

Results and Discussion

1. ***Supplemental Figure S1-S28.. P7-32.***
2. ***Supplemental Table S1.. P16.***
3. ***Supplemental Table S2.. P32.***
4. ***References.. P33.***

1. Materials and chemicals

Iodine (I_2) was acquired from Aladdin Industrial Co. Urea (CN_2H_4O), sodium hypophosphite monohydrate ($NaH_2PO_2.H_2O$), nickel nitrate hexahydrate ($Ni(NO_3)_2 \cdot 6H_2O$), ammonia fluoride (NH_4F) , and ethanol (C_2H_5OH) were bought from Sinopharm Holdings Chemical Reagent Co. Nickel foam (NF) was purchased from Kunshan Guangjiayuan New Material Co. During the experiment all water used was ultrapure water (18.2 MΩ cm^{-1}). No further purification of all chemicals was done and they were used directly.

2. Synthesis methods

2.1 Synthesis of 1.2 $Ni(OH)_2$ /NF precursor. Firstly, the nickel foam was pretreated to remove the oil and oxide layer on its surface. Ultrasonic cleaning was carried out with 4M hydrochloric acid, acetone, ethanol and distilled water successively, and dried under Ar gas high-speed airflow. 0.58g $Ni(NO3)_2.6H_2O$, 0.15g NH_4F and 0.60g urea were dissolved in 30mL deionized water and stirred vigorously for 20min. Then the mixture was transferred to a autoclave liner. Nickel foam with a size of 2 cm x 3 cm is immersed in the autoclave liner for hydrothermal synthesis. The autoclave was treated at 120℃ for 10 hours. After cooling to room temperature, a distinct light green substance appeared on the surface of the nickel foam, which was removed and cleaned with deionized water and ethanol to remove adsorbed impurities on the surface. Finally, it was dried for 15 hours in a vacuum environment at 60℃.

2.2 Synthesis of $Ni_5P_{4-X}I_X/Ni_2P$/NF: The $Ni(OH)_2$/NF precursor was placed in a tubular furnace with 0.4g $NaH_2PO_2.H_2O$ in the middle stream and 0.6g I_2 in the upper stream. The carrier gas was argon. The precursor was heat treated at 350℃ for 2h and then removed after natural cooling to room temperature. The corresponding $Ni_5P_{4-X}I_X/Ni_2P$ -250, $Ni_5P_{4-X}I_X/Ni_2P$-450 and $Ni_5P_{4-X}I_X/Ni_2P$ -550 samples were obtained at 250℃, 450℃ and 550℃, respectively.

2.3 Synthesis of $Ni(OH)_2$@I/NF and Ni_2P/NF: The $Ni(OH)_2$/NF precursor was also placed downstream of the tubular furnace under reaction conditions similar to that of $Ni_5P_{4-X}I_X/Ni_2P$. The difference is that 0.4 g of $NaHPO_2.H_2O$ and 0.6g I_2 were respectively used as upstream raw materials for 2 hours of heat treatment at 350℃.

3. Materials Characterisation

X-ray diffraction (XRD) was distributed employing a D8-tools Bragg-Brentano diffractometer from germany with a conductor Kα emission supply (λ = 0.15418 nm). Scanning electron microscope (SEM) images were obtained by a Hitachi SU8010 scanning electron microscope taken at 10 kV. Transmission electron microscopy (TEM), high-resolution transmission electron microscopy (HRTEM) and selected area electron diffraction (SAED) were acquired by using a JEOL JEM-2000F instrument from Japan. X-ray photoelectron spectroscopy (XPS) measurements were gained by testing using an ESCALAB-250 instrument (Thermo Fisher Scientific, USA) with a 0.1 eV energy resolution hemispherical detector and a monochromatic Al-Kα (1486.6 eV) radiation source. Electron Paramagnetic Resonance (EPR) spectroscopy was obtained by using an EPR JES-FA200 electron paramagnetic resonance spectrometer. At the Beijing Synchrotron Radiation Facility (BSRF) 1W1B beamline station in China, X-ray absorption near edge structure (XANES) spectra of the nickel K-edge were measured in transmission mode. The obtained data are processed with the ATHENA module of the IFEFFIT package and wavelet transformed with Fortran through the Morlet function. Surface wettability tests were performed at room temperature using a contact angle analyzer (Kruss DSA 30).

4. Electrochemical Measurements

All electrochemical tests were performed in 1 M KOH solution using a CHI 760E electrochemical workstation (Shanghai Chenhua). The classical three-electrode system is used for electrochemical HER and OER performance testing, where a cut material is installed on the electrode clamp directly as the working electrode, Hg/HgO as the reference electrode, and a carbon rod as the counter electrode. All electrochemical potentials in this work are relative to the reversible hydrogen electrode (RHE) and are given by the following equation. $E_{(RHE)} = E_{(Hg/HgO)} + 0.098$ V + 0.059 x pH. The HER and OER polarization curves were obtained by linear voltammetry (LSV) technique at 2 mV s^{-1} and corrected using the automatic iR compensation function of the workstation. The electrochemical impedance spectra (EIS) were obtained in the frequency range of 100000-0.01 Hz with an AC amplitude of 5 mV. The electrochemical active area was compared by cyclic voltammetry (CV) tests to obtain the electrochemical double layer capacitance (C_{dl}) in the corresponding potential range of HER and OER. Stepped currents were used to test electrochemical stability. Two electrode cells were used throughout the water splitting experiments, where $Ni_5P_{4-x}I_x/Ni_2P$/NF was used as positive and negative electrodes.

5. DFT calculations

All first-principles calculations were based on density-functional theory (DFT), as implemented in the Vienna ab initio simulation package codes (VASP). The electrons and ions interactions were described by the projector augmented wave (PAW) potential. The generalized gradient approximation (GGA) formulated by Perdew-Burke-Ernzerhof (PBE) form as the exchange-correlation functional. The kinetic energy cutoff for the plane wave basis was chosen to be 500 eV. The local minimum is obtained with convergence of the energy was set to be less than 10^{-5} eV and the tolerance of atomic force was less than -0.05 eV/Å. For bulk materials, k-point grid spacing set to $2\pi \times 0.03$ Å^{-1} for electronic Brillouin zone integration. The

Cohesive energy (E_{coh}) was defined as: $E_{coh} = E_{bulk}/N - E_{at}$, in which E_{bulk} represents the energy of the bulk unit cell containing N atoms and E_{at} is the energy of the isolated atom in a vacuum. Ni_2P is the hexagonal structure (P321 (150)), Ni_5P_4 and $Ni_5P_{4-x}I_x$ are also hexagonal structure (P63mc (186)). The infrastructure comes from the Inorganic Crystal Structure Database (ICSD). The (111) and (212) surfaces of Ni_2P and $N_{i5}P_4$ were chosen for the study, respectively. The surface was selected according to the results of pre-experimental characterization, and the selected surface was consistent with the results of XRD and HRTEM. Van der Waals (vdW) interactions were considered by Grimme's scheme (DFT+D3) method. The k-points were set to 3×3×1 with the Monkhorst-Pack mesh was utilized for the surfaces computations. To avoid interactions between periodic images, the vacuum space was set to 15 Å in the z-direction. Furthermore, the thermodynamic free energies of each elementary step in the whole HER process were determined by the computational hydrogen electrode (CHE) technique proposed by Nørskov *et al.* [1] According to this model, the changes in Gibbs free energy (ΔG) for all electrochemical steps was defined as: $\Delta G = \Delta E + \Delta E_{ZPE} - T\Delta S$, where the reaction energy (ΔE) can be directly obtained by analyzing the DFT total energies. The zero-point energy difference (ΔE_{ZPE}) between the products and the reactants can be computed from the vibrational frequencies. ΔS is the change in entropy between the products and the reactants at room temperature ($T = 298.15\ K$). The active sites of HER and OER are Ni sites on the surface.

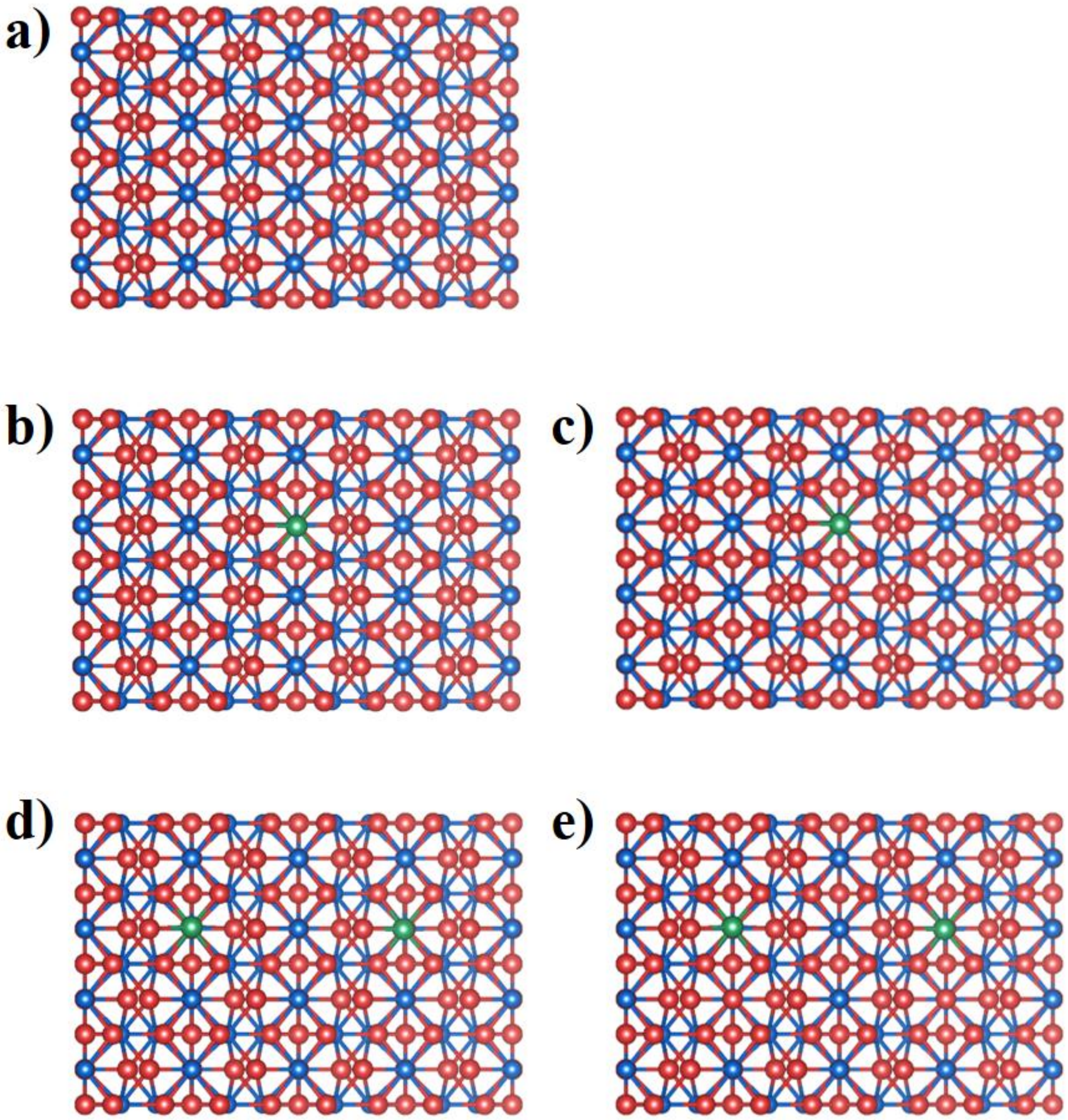

Figure S1. Ni_2P bulk structure based atomic structure models for doping and defect states corresponding to a) Pristine, b) I-doped, c) I-doped with 1 P vacancy (1V), d) 2I-doped, e) 2I-doped with 2 P vacancies (2V).

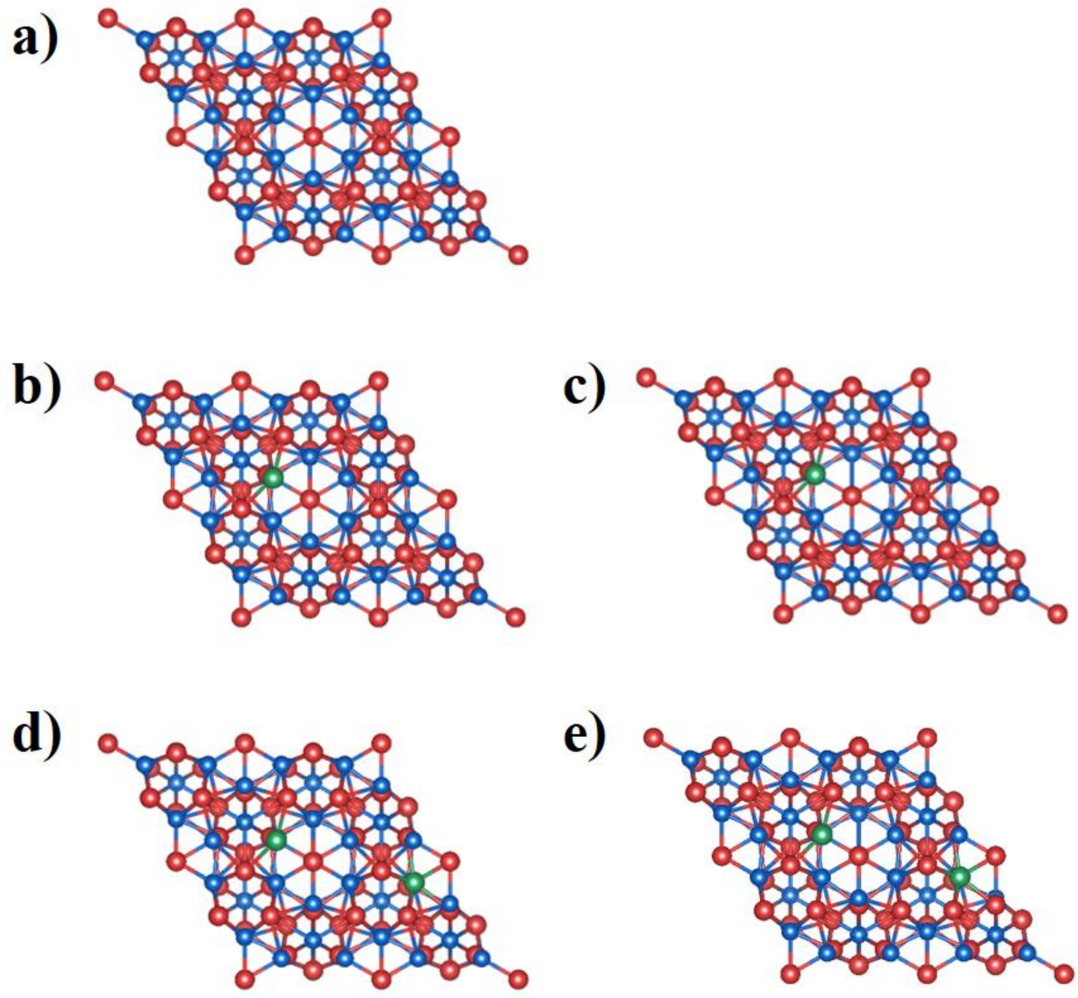

Figure S2. Ni_5P_4 bulk structure based atomic structure models for doping and defect states corresponding to a) Pristine, b) I-doped, c) I-doped with 1 P vacancy (1V), d) 2I-doped, e) 2I-doped with 2 P vacancies (2V).

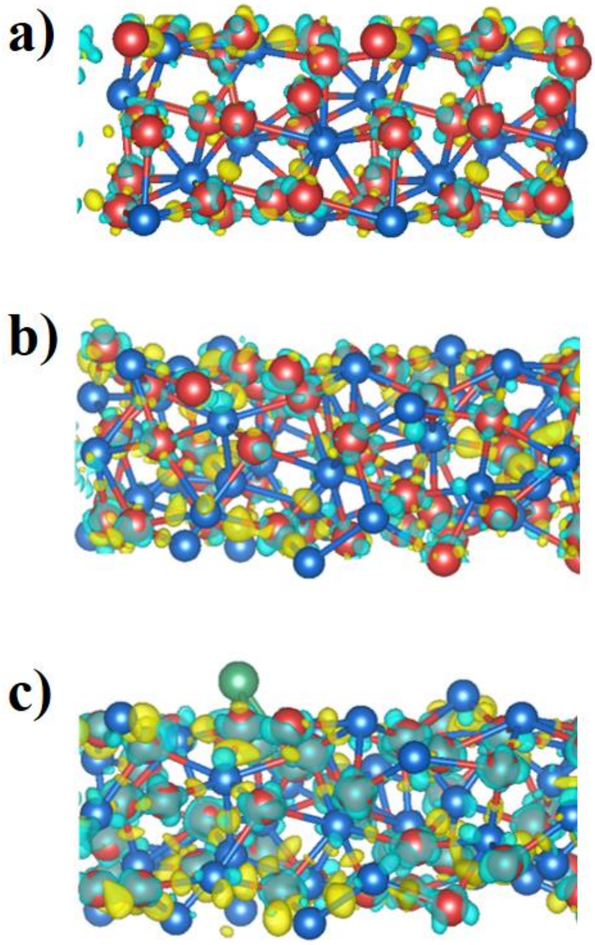

Figure S3. Charge density difference of a) Ni_2P, (b) Ni_5P_4, (c) $Ni_5P_{4-x}I_x$ structure surface.

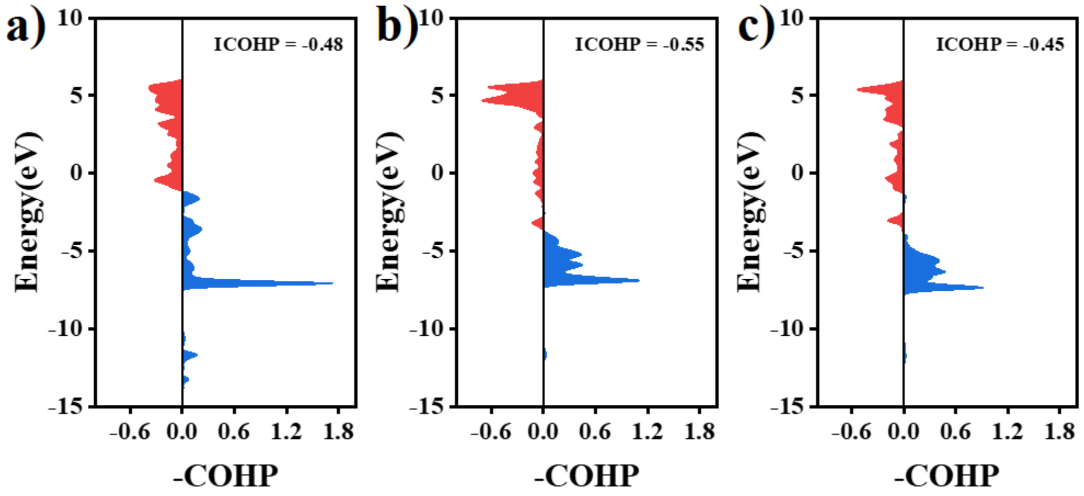

Figure S4. COHP analysis of a) Ni_2P, b) Ni_5P_4 and c) $Ni_5P_{4-x}I_x$, red for antibonding, blue for bonding.

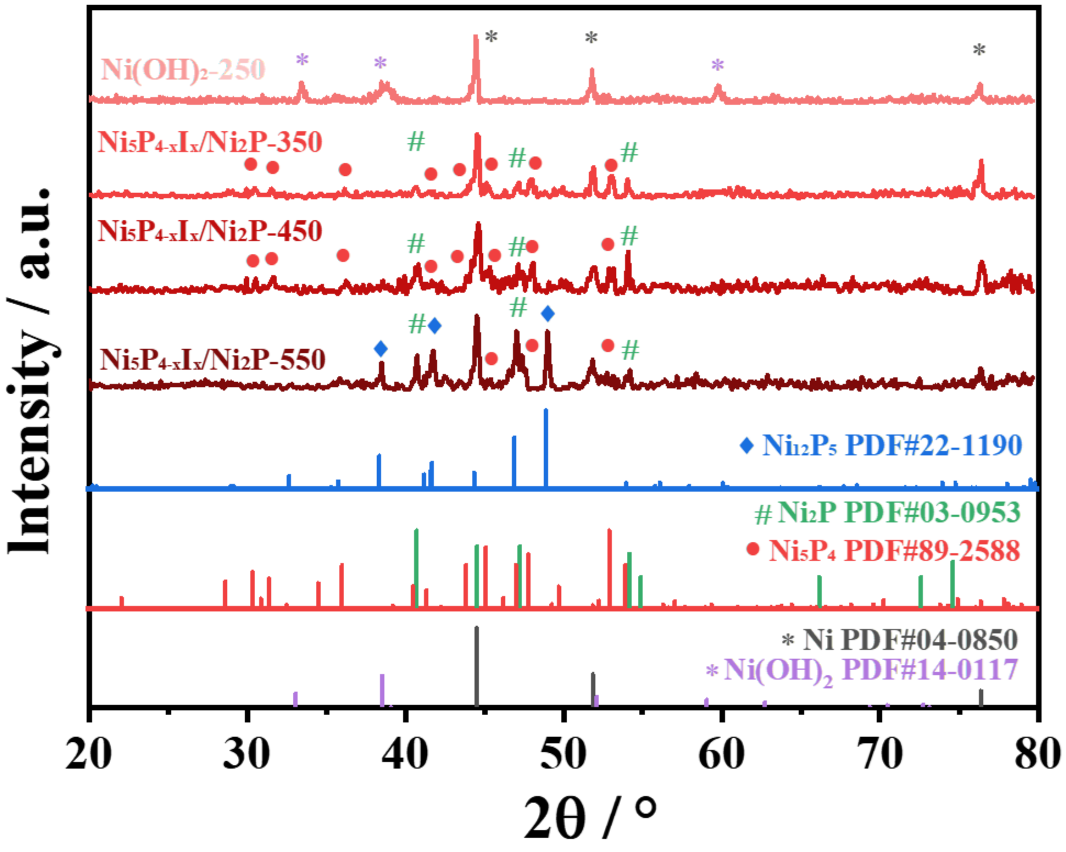

Figure S5. XRD patterns of $Ni_5P_{4-x}I_x/Ni_2P$ synthesized at different temperatures corresponding to 250 ℃, 350 ℃, 450 ℃, and 550 ℃.

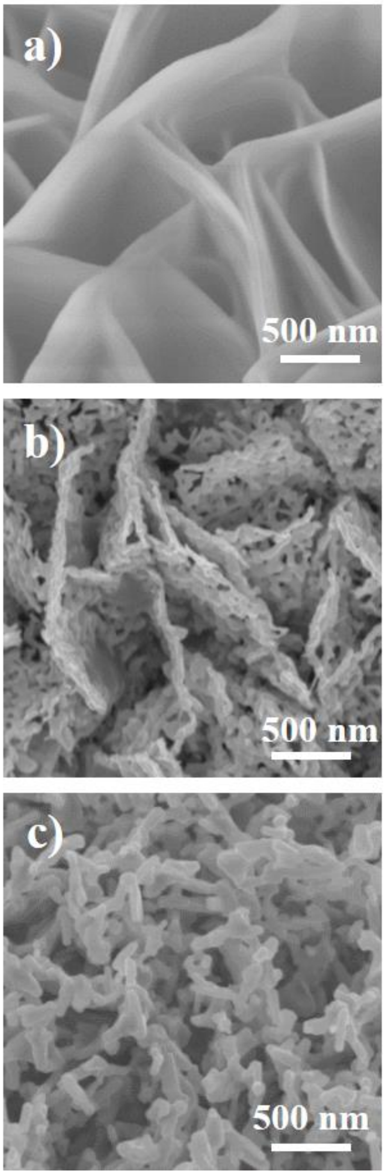

Figure S6. SEM images of (a) $Ni(OH)_2$, (b) Ni_2P, and (c) $Ni_5P_{4-x}I_x/Ni_2P$.

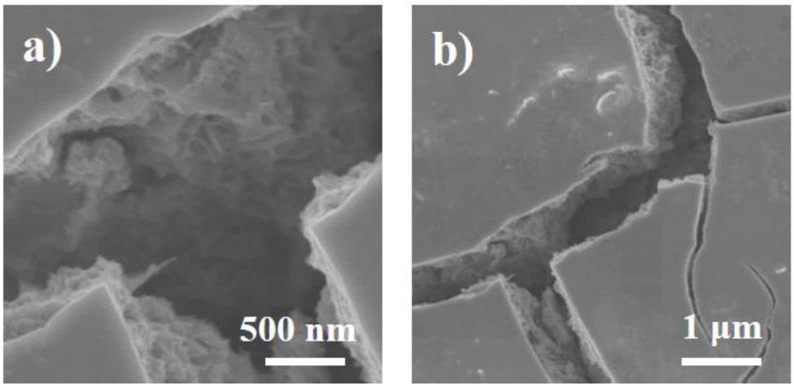

Figure S7. (a) SEM images of $Ni(OH)_2$@I, (b) Corresponding low magnification image.

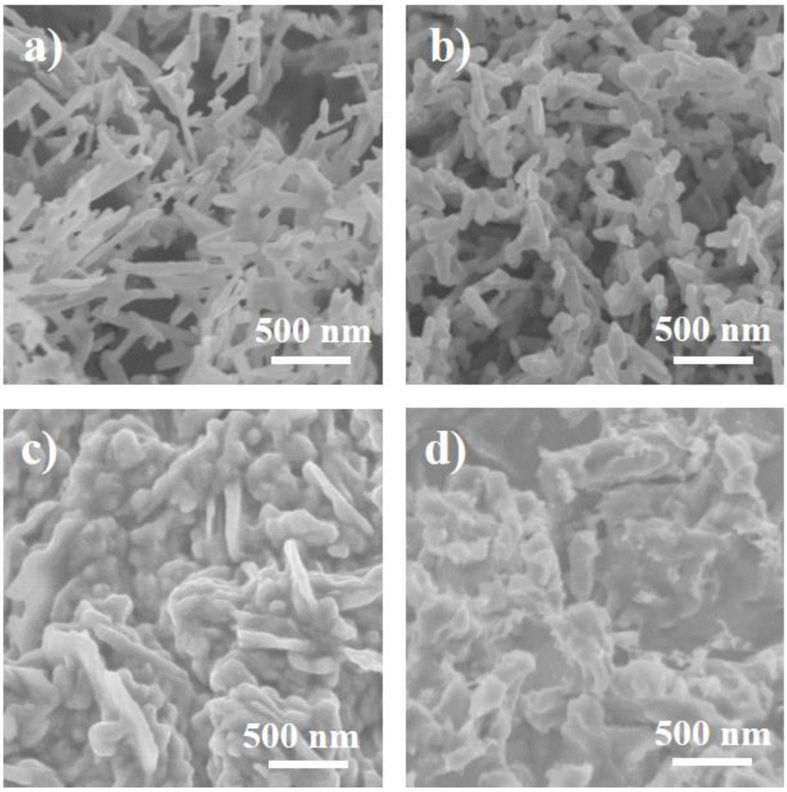

Figure S8. SEM images of $Ni_5P_{4-x}I_x/Ni_2P$ at different synthesis temperatures are shown for comparison at a) 250 ℃, b) 350 ℃, c) 450 ℃, and d) 550 ℃.

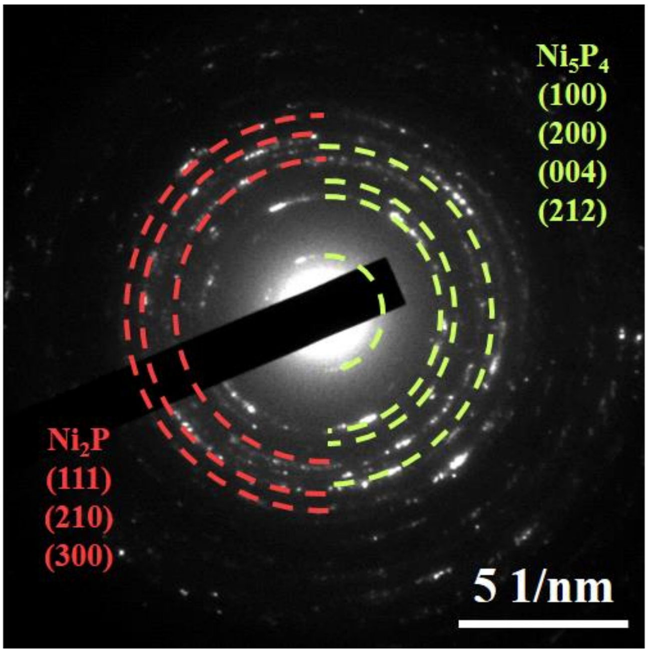

Figure S9. The selected area electron diffraction (SAED) pattern of $Ni_5P_{4-x}I_x/Ni_2P$.

.

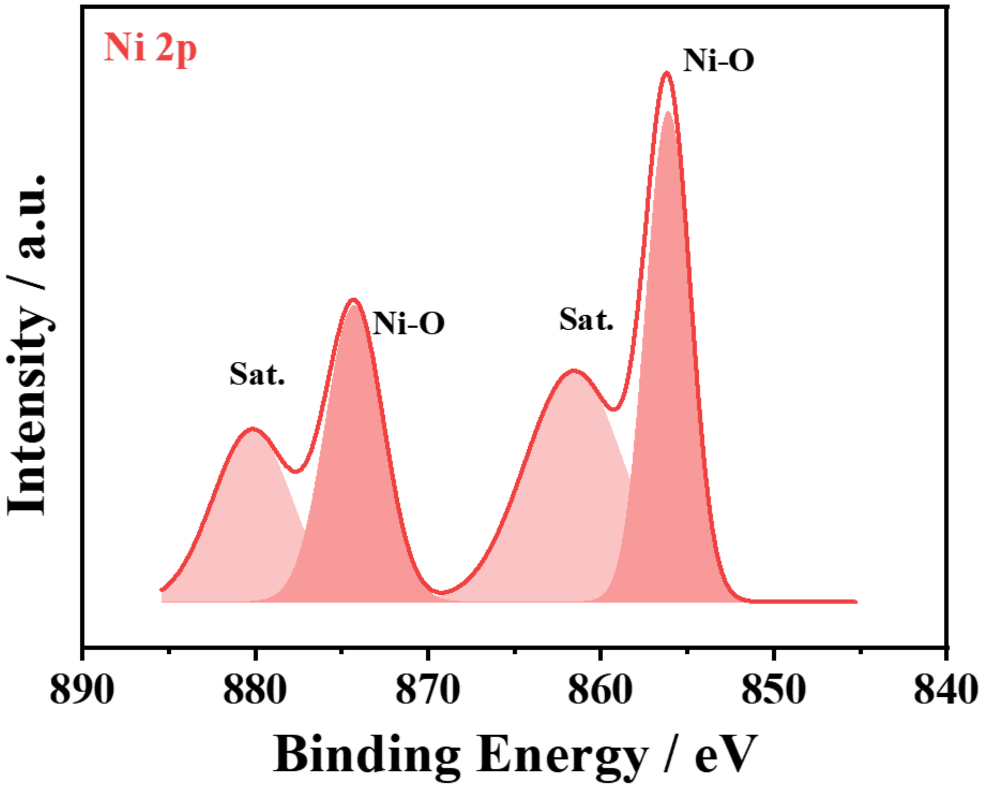

Figure S10. High-resolution XPS spectra of Ni 2p in $Ni(OH)_2$@I

	Ni_2P	$Ni_5P_{4-x}I_x/Ni_2P$
Ni	68.12%	43.67%
P	31.88%	51.19%
I	/	5.14%

Table S1. XPS surface atomic percent of Ni_2P and $Ni_5P_{4-x}I_x/Ni_2P$.

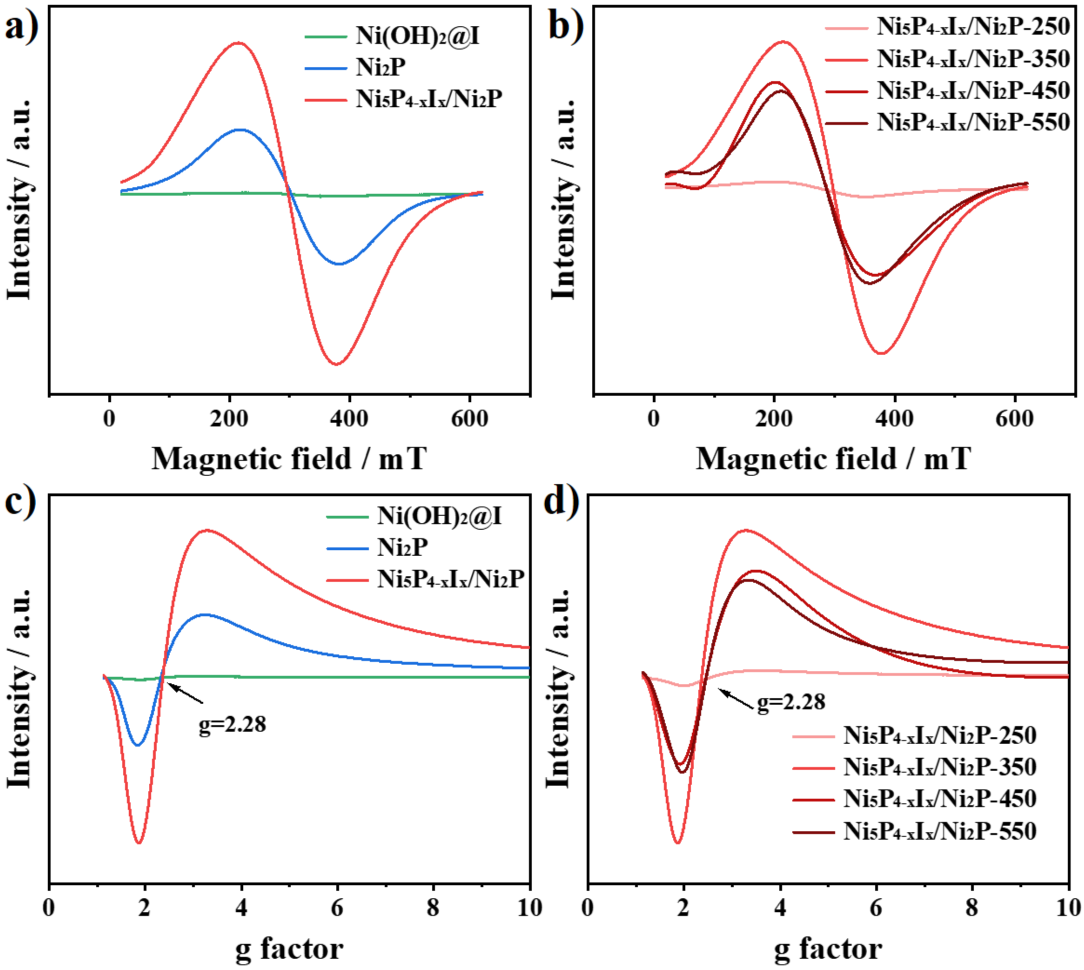

Figure S11. a) EPR spectra of $Ni(OH)_2$@I, Ni_2P and $Ni_5P_{4-x}I_x/Ni_2P$, b) EPR spectra of $Ni_5P_{4-x}I_x/Ni_2P$ at different synthesis temperatures are shown for comparison at 250 ℃, 350 ℃, 450 ℃ and 550 ℃, c) g-factor transformation format of a image, d) g-factor transformation format of b image.

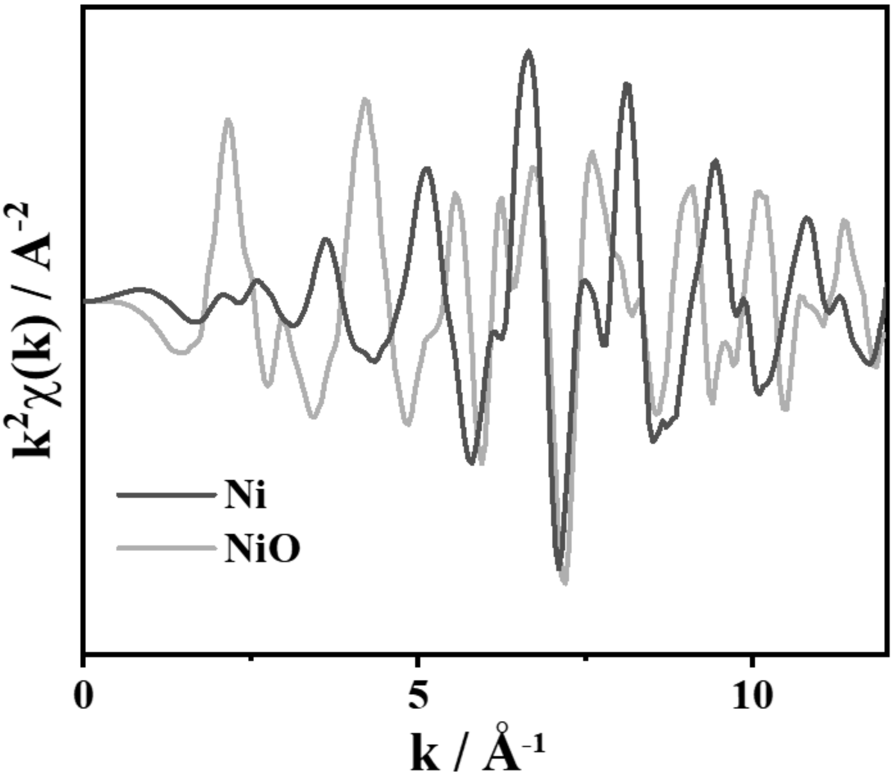

Figure S12. Ni K-edge extended XANES oscillation functions $k^2\chi(k)$ of Ni foil and NiO.

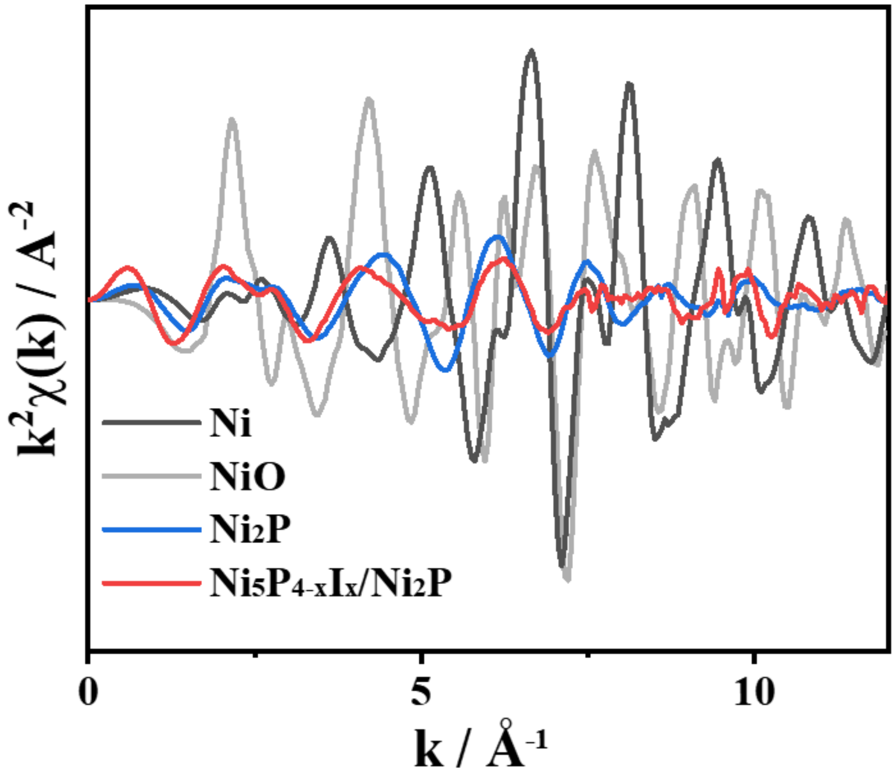

Figure S13. Ni K-edge extended XANES oscillation functions $k^2\chi(k)$ of $Ni_5P_{4-x}I_x/Ni_2P$ and Ni_2P, compared with Ni foil and NiO.

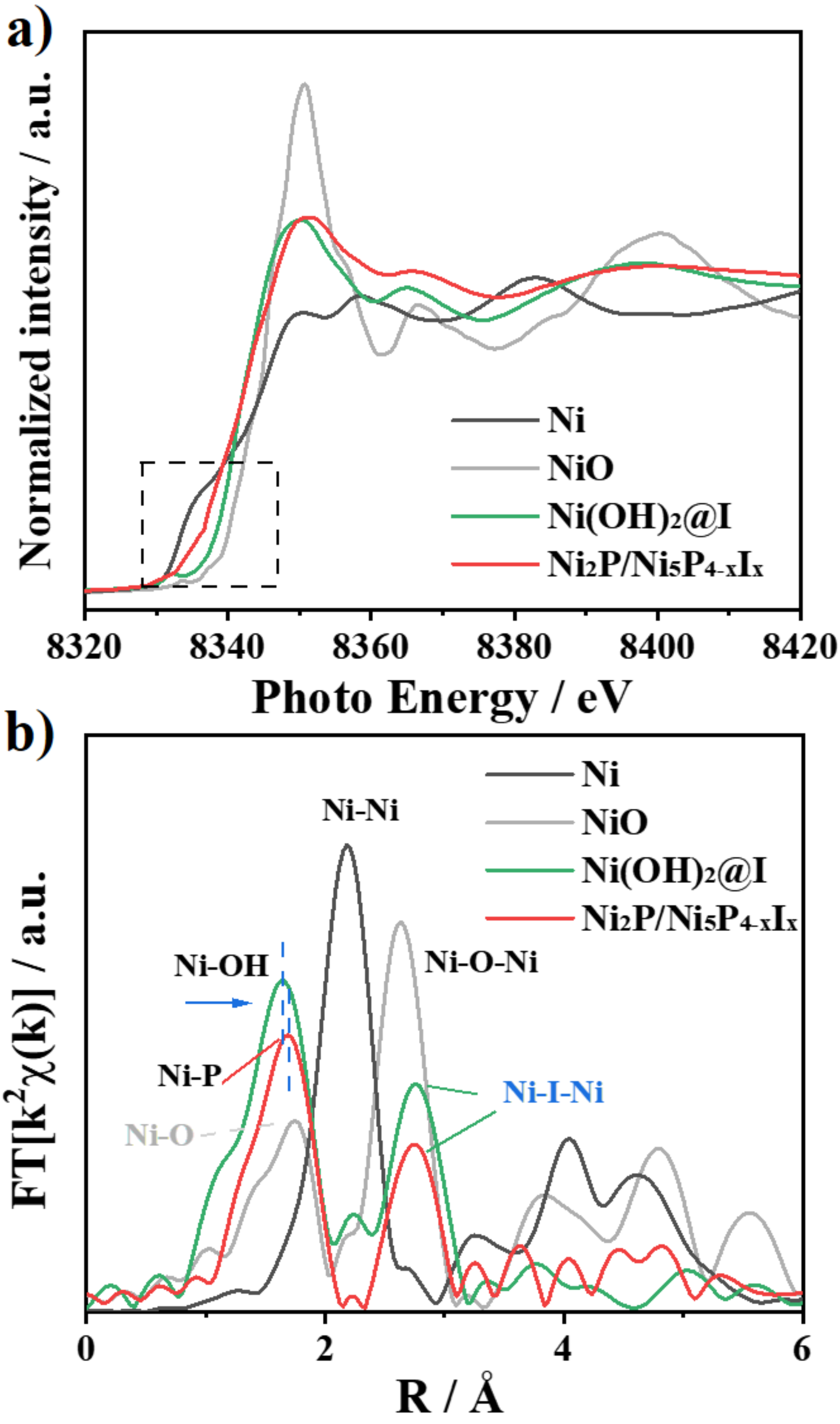

Figure S14. The normalized XANES spectra at the Ni K-edge of $Ni(OH)_2$@I, compared with Ni foil, NiO and $Ni_5P_{4-x}I_x/Ni_2P$.

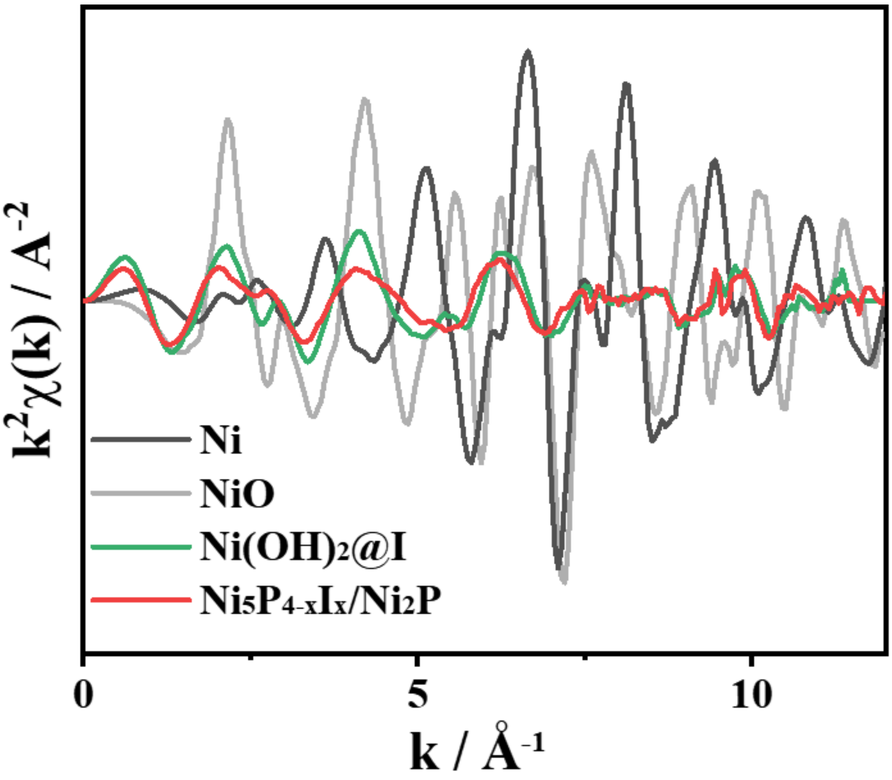

Figure S15. Ni K-edge extended XANES oscillation functions $k^2\chi(k)$ of $Ni_5P_{4-x}I_x/Ni_2P$ and $Ni(OH)_2$@I, compared with Ni foil and NiO.

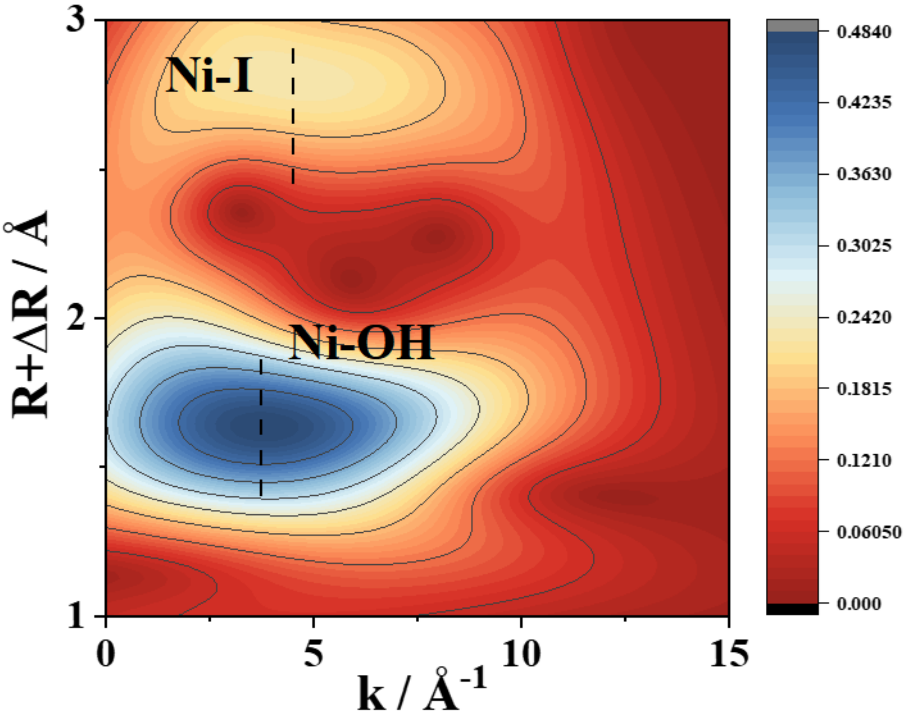

Figure S16. Wavelet transformed plots for the k^2-weighted EXAFS signals of the Ni K-edge of $Ni(OH)_2$@I.

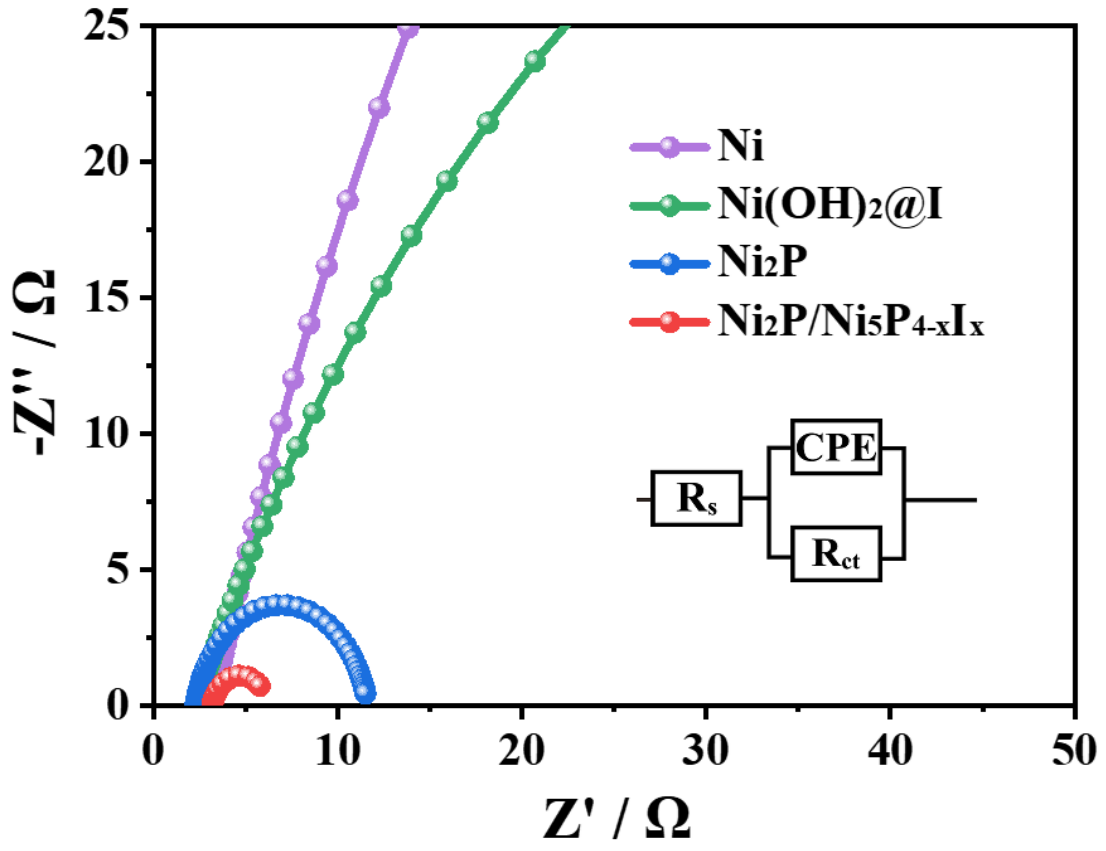

Figure S17. Electrochemical impedance spectroscopy (EIS) Nyquist plots during the HER for Ni, $Ni(OH)_2$@I, Ni_2P and $Ni_5P_{4-x}I_x/Ni_2P$ (Inset: the equivalent circuit model).

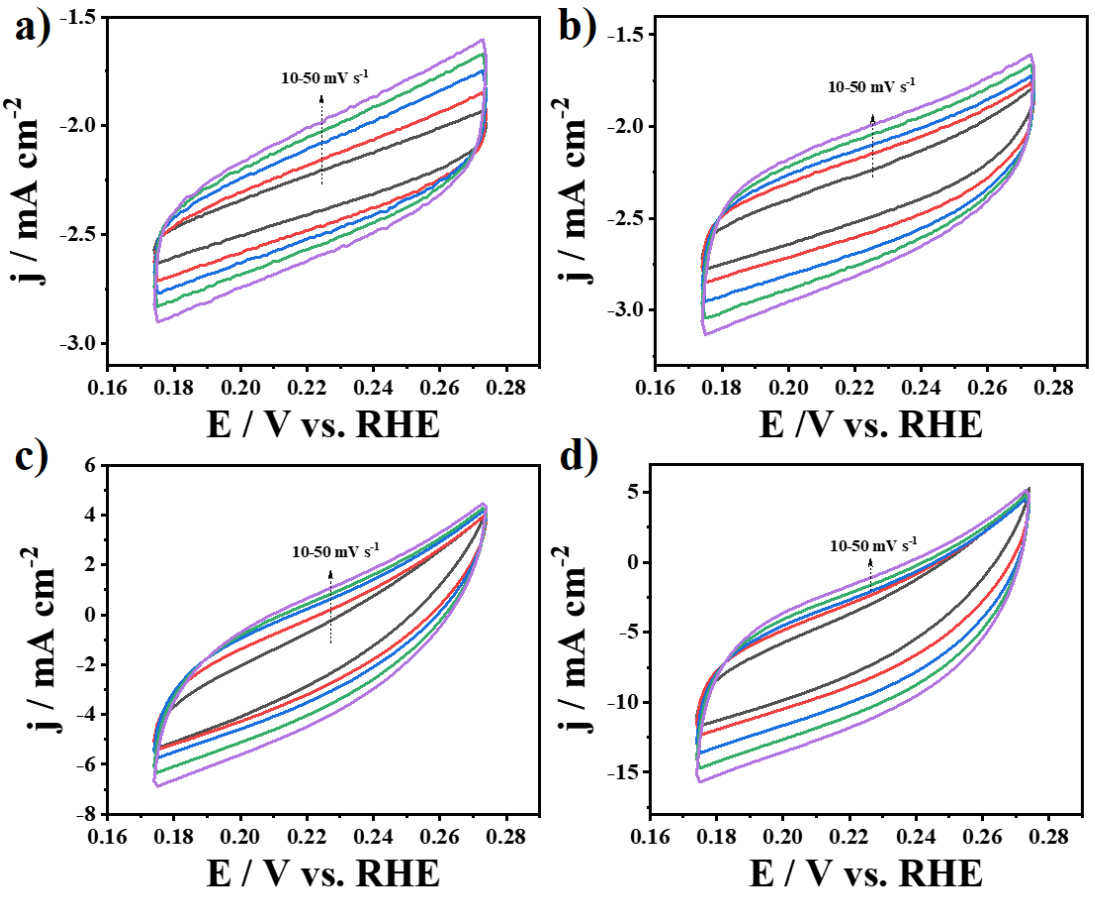

Figure S18. Cyclic voltammetry curves of a) Ni, b) $Ni(OH)_2$@I, c) Ni_2P and d) $Ni_5P_{4-x}I_x/Ni_2P$ in 1M KOH for HER at the scan rates from 10 mV s^{-1} to 50 mV s^{-1}.

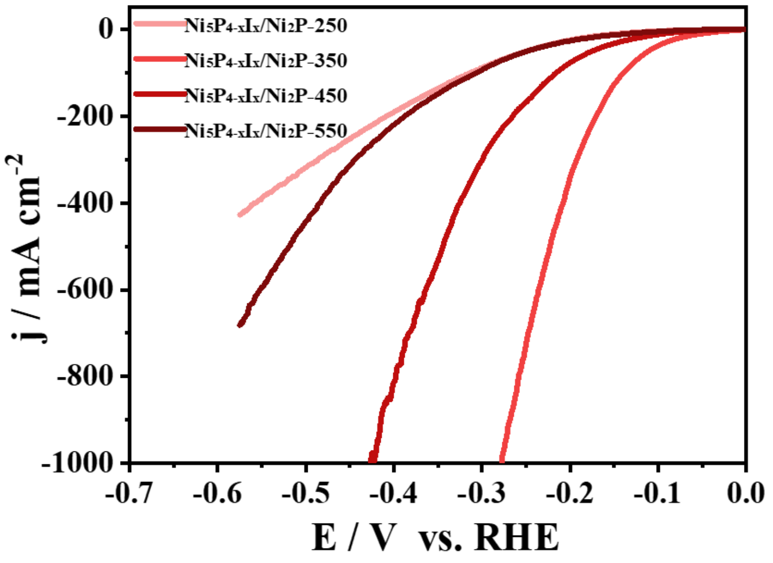

Figure S19. Hydrogen evolution reaction electrocatalysis in 1 M KOH. IR-corrected polarization curves per geometric area of $Ni_5P_{4-x}I_x/Ni_2P$ at different synthesis temperatures are shown for comparison at 250 ℃, 350 ℃, 450 ℃, and 550 ℃.

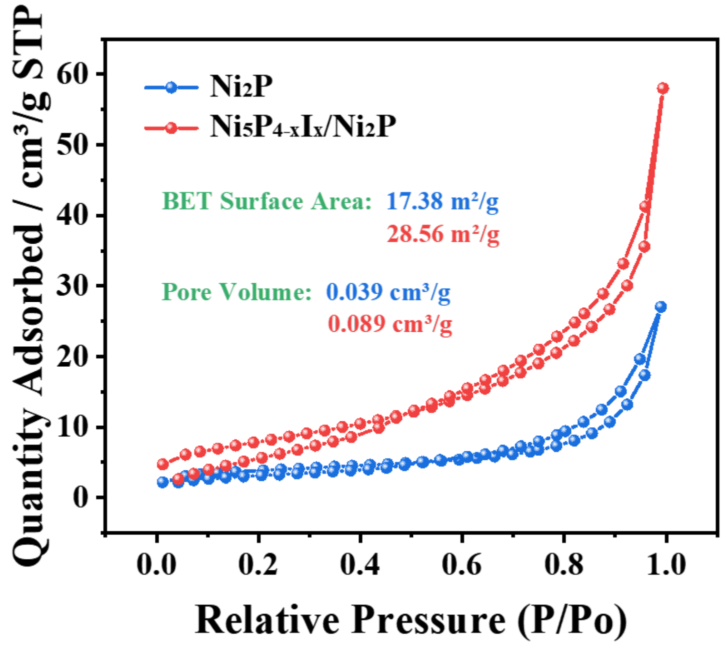

Figure S20. N_2 adsorption and desorption curve of $Ni_5P_{4-x}I_x/Ni_2P$ and Ni_2P.

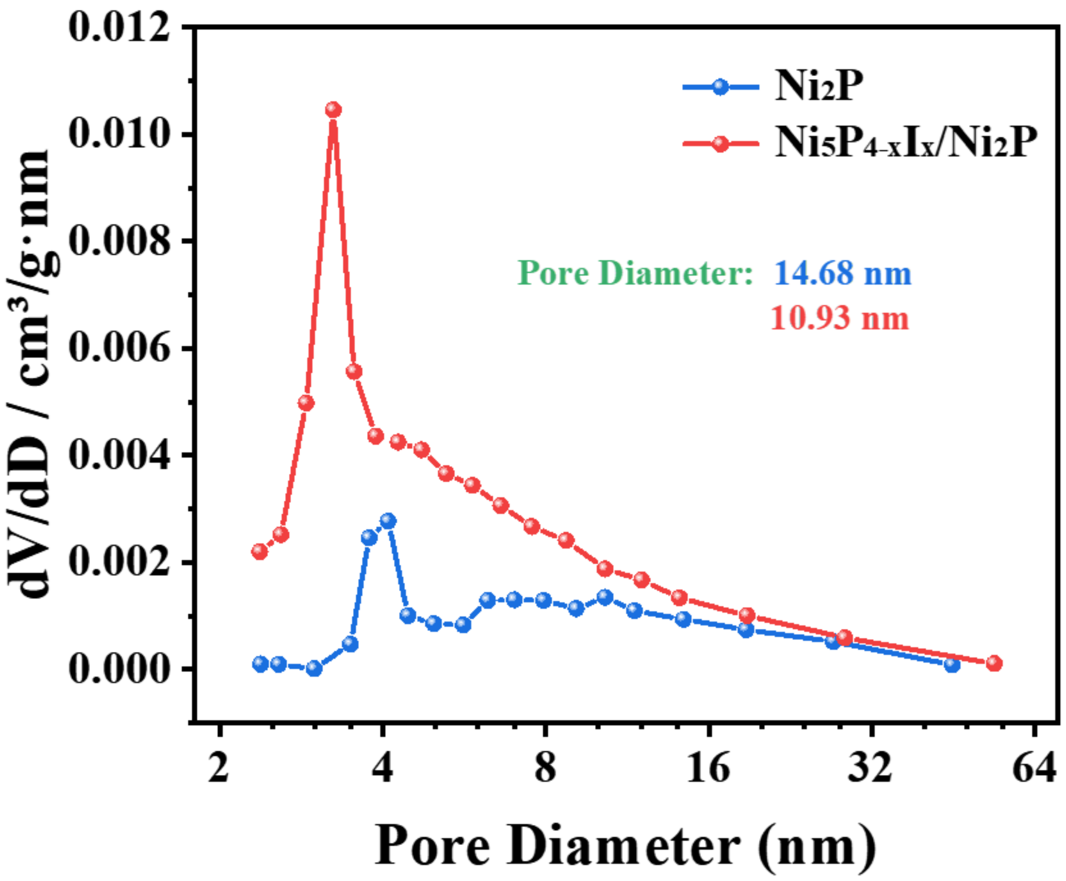

Figure S21. Pore size distribution of $Ni_5P_{4-x}I_x/Ni_2P$ and Ni_2P.

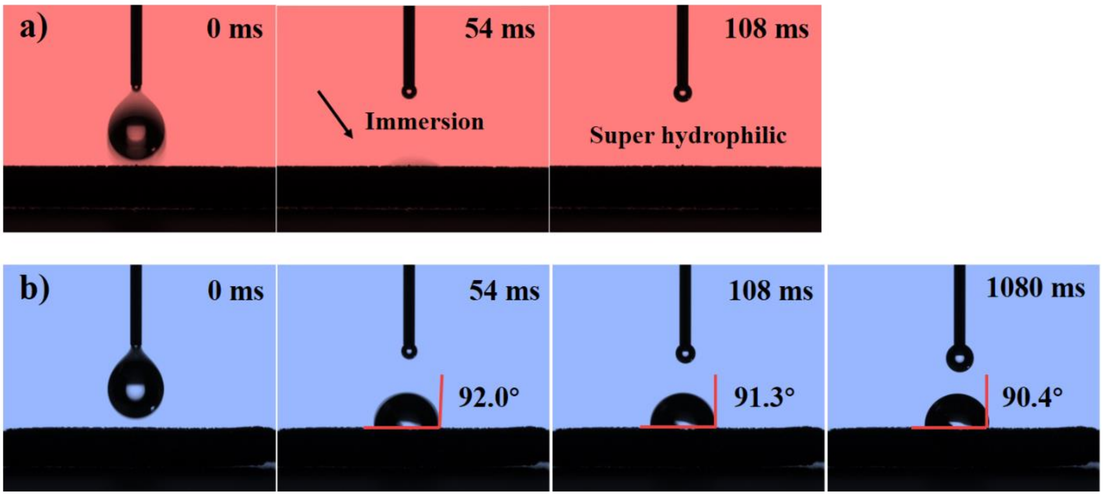

Figure S22. Pictures of the change in contact angle over time in a wettability test of a) $Ni_5P_{4-x}I_x/Ni_2P$ and b) Ni_2P.

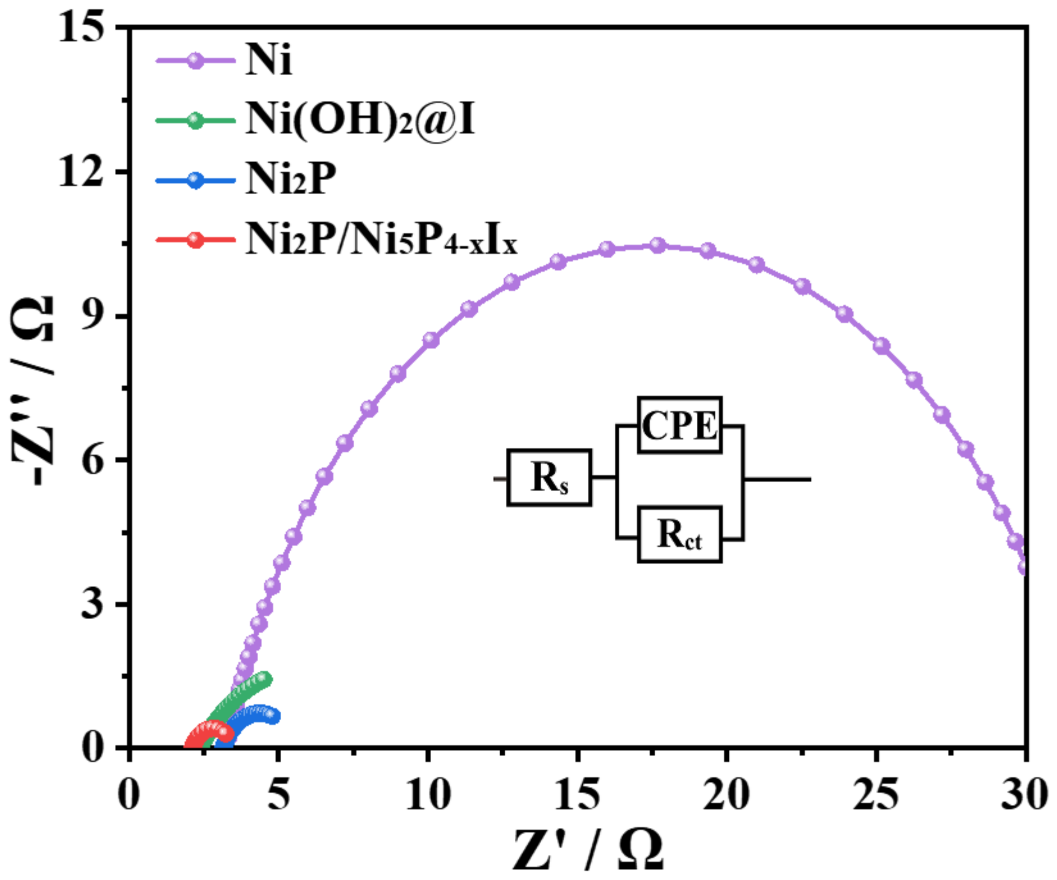

Figure S23. Electrochemical impedance spectroscopy (EIS) Nyquist plots during the OER for Ni, $Ni(OH)_2$@I, Ni_2P and $Ni_5P_{4-x}I_x/Ni_2P$ (Inset: the equivalent circuit model).

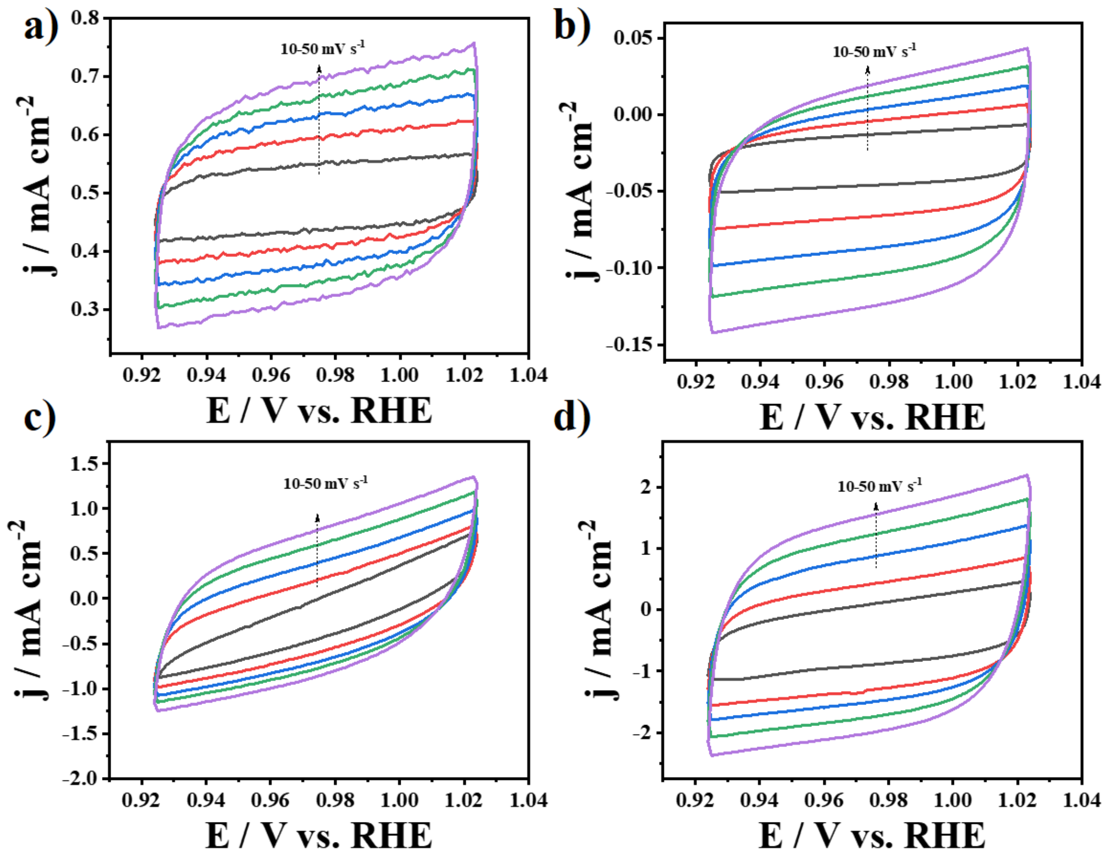

Figure S24. Cyclic voltammetry curves of a) Ni, b) $Ni(OH)_2$@I, c) Ni_2P and d) $Ni_5P_{4-x}I_x/Ni_2P$ in 1M KOH for OER at the scan rates from 10 mV s^{-1} to 50 mV s^{-1}.

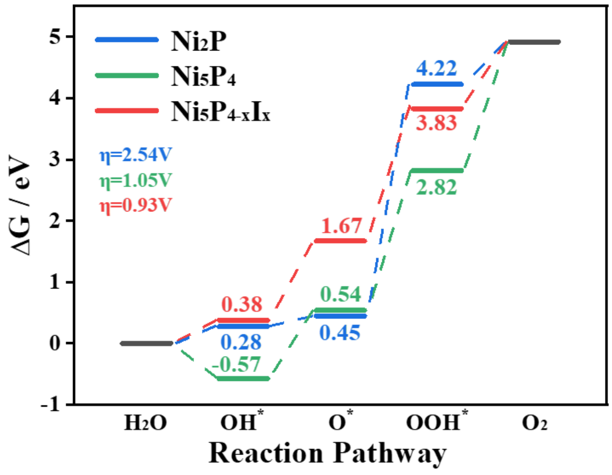

Figure S25. OER free energies of Ni_2P, Ni_5P_4 and $Ni_5P_{4-x}I_x$

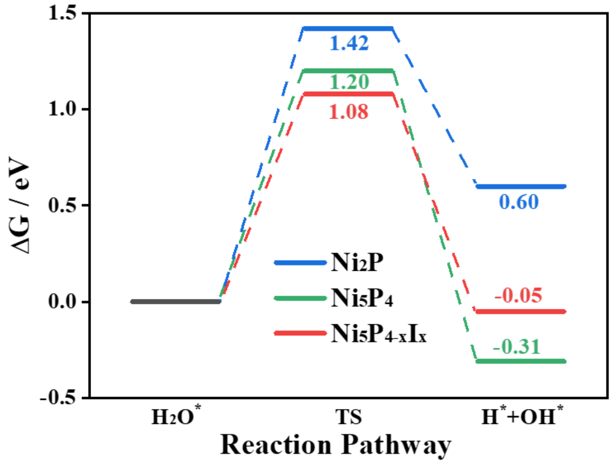

Figure S26. Dissociation energy of water during alkaline HER for Ni_2P, Ni_5P_4 and $Ni_5P_{4-x}I_x$

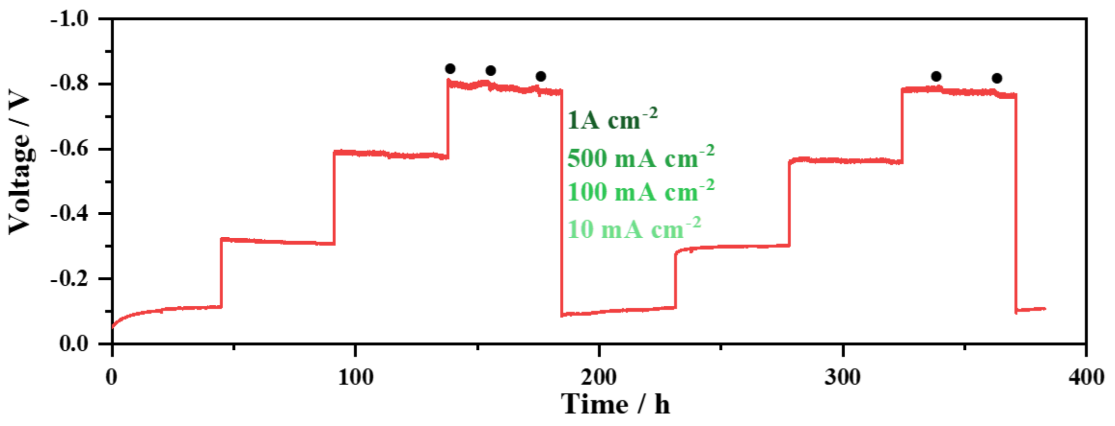

Figure S27. Long-term HER stability test for $Ni_5P_{4-x}I_x/Ni_2P$ at step current densities of 10,100, 500 and 1000 mA cm^{-2}.

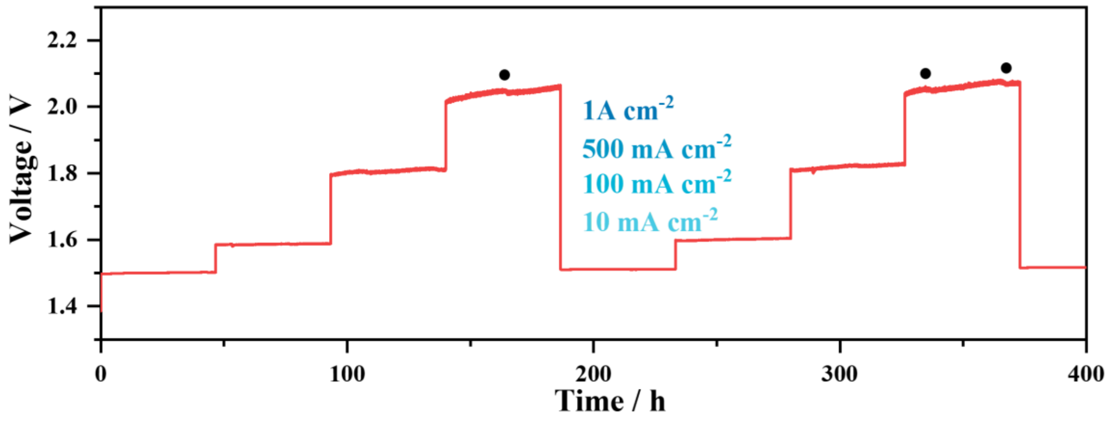

Figure S28. Long-term OER stability tests for $Ni_5P_{4-x}I_x/Ni_2P$ at step current densities of 10,100, 500 and 1000 mA cm^{-2}.

Electrolyte	Materials	HER	OER	OWS	References
		η10 (mV)	η (mV)	η 10(V)	
1 M KOH	**Fe-Ni_2P@PC/Cu_xS**	113	50~330	1.62	[2]
	**VPO/Ni_2P**	154	-	-	[3]
	**NiMnOP**	189	10~191	1.51	[4]
	**CoP_3/Ni_2P**	57.3	100~323	1.557	[5]
	**Ni_2P-Fe_2P**	41	10~218	1.56	[6]
	**p-NFNR@Ni− Co−P**	193	10~272	1.62	[7]
	**NiFeSP**	91	50~240	1.58	[8]
	**Fe-Ni_2P**	33	10~205	100~1.57	[9]
	**Ni_2P/Ni_3S_2**	129	10~141	1.50	[10]
	**Ni_2P–$Ni_{12}P_5$**	76	-	-	[11]
	**CoP/Ni_5P_4/CoP**	71	-	-	[12]
	**Ni_2P–Fe_2P–Ru_2P**	78.6	10~195	1.49	[13]
	**S-NiFeP,**	48	10~201	1.50	[14]
	**This work**	**45**	**10~163**	**1.46**	

Table S2: Comparison of the electrocatalytic performance of recently reported nickel phosphide modified electrocatalysts in alkaline media.

Supplementary References:

[1] a) H. J. Jeong, K. K. Kim, S. Y. Jeong, H. Min, Y. H. Lee, *J. Phys. Chem. B* **2004**, 108, 17886; b) J. Rossmeisl, A. Logadottir, J. K. Nørskov, *Chem. Phys.* **2005**, 319, 178.

[2] D. T. Tran, H. T. Le, V. H. Hoa, N. H. Kim, J. H. Lee, *Nano Energy* **2021**, 84, 105861.

[3] W. Xu, Q. Li, W. Zhong, B. Sun, Q. Huang, X. Nan, Y. Gao, Y. Yang, Q. Zhang, N. Yang, X. Li, *J. Energy Chem.* **2022**, 65, 674.

[4] J. Balamurugan, T. T. Nguyen, V. Aravindan, N. H. Kim, J. H. Lee, *Nano Energy* **2020**, 69, 104432.

[5] J. Zhang, H. Zhou, Y. Liu, J. Zhang, Y. Cui, J. Li, J. Lian, G. Wang, Q. Jiang, *ACS Appl. Mater. Interfaces* **2021**, 13, 52598.

[6] L. Wu, L. Yu, F. Zhang, B. McElhenny, D. Luo, A. Karim, S. Chen, Z. Ren, *Adv. Funct. Mater.* **2020**, 31, 2006484.

[7] Y. Feng, R. Wang, P. Dong, X. Wang, W. Feng, J. Chen, L. Cao, L. Feng, C. He, J. Huang, *ACS Appl. Mater. Interfaces* **2021**, 13, 48949.

[8] Y. Xin, X. Kan, L. Y. Gan, Z. Zhang, *ACS Nano* **2017**, 11, 10303.

[9] H. Sun, Y. Min, W. Yang, Y. Lian, L. Lin, K. Feng, Z. Deng, M. Chen, J. Zhong, L. Xu, Y. Peng, *ACS Catal.* **2019**, 9, 8882.

[10] W. Z. Chen, P. Y. Liu, L. Zhang, Y. Liu, Z. L. Liu, J. L. He, Y. Q. Wang, *Chem. Eng. J.* **2021**, 424, 130434.

[11] Z. Wang, S. Wang, L. Ma, Y. Guo, J. Sun, N. Zhang, R. Jiang, *Small* **2021**, 17, e2006770.

[12] I. K. Mishra, H. Q. Zhou, J. Y. Sun, F. Qin, K. Dahal, J. M. Bao, S. Chen, Z. F. Ren, *Energy Environ. Sci.* **2018**, 11, 2246.

[13] S.-H. Cai, X.-N. Chen, M.-J. Huang, J.-Y. Han, Y.-W. Zhou, J.-S. Li, *J. Mater. Chem. A* **2022**, 10, 772.

[14] S. S. Li, L. Wang, H. Su, A. N. Hong, Y. X. Wang, H. J. Yang, L. Ge, W. Y. Song, J. Liu, T. Y. Ma, X. H. Bu, P. Y. Feng, *Adv. Funct. Mater.* **2022**, 2200733.
